# Depletion of Host Cell Focal Adhesion Kinase Increases the Susceptibility to Invasion by *Trypanosoma cruzi* Metacyclic Forms

**DOI:** 10.3389/fcimb.2019.00231

**Published:** 2019-06-26

**Authors:** Thiago Souza Onofre, João Paulo Ferreira Rodrigues, Nobuko Yoshida

**Affiliations:** Departamento de Microbiologia, Imunologia e Parasitologia, Escola Paulista de Medicina, Universidade Federal de São Paulo, São Paulo, Brazil

**Keywords:** *Trypanosoma cruzi*, metacyclic trypomastigote, host cell invasion, focal adhesion kinase, actin cytoskeleton, lysosome distribution

## Abstract

Focal adhesion kinase (FAK), a cytoplasmic protein tyrosine kinase (PTK), is implicated in diverse cellular processes, including the regulation of F-actin dynamics. Host cell F-actin rearrangement is critical for invasion of *Trypanosoma cruzi*, the protozoan parasite that causes Chagas disease. It is unknown whether FAK is involved in the internalization process of metacyclic trypomastigote (MT), the parasite form that is important for vectorial transmission. MT can enter the mammalian host through the ocular mucosa, lesion in the skin, or by the oral route. Oral infection by MT is currently a mode of transmission responsible for outbreaks of acute Chagas disease. Here we addressed the question by generating HeLa cell lines deficient in FAK. Host cell invasion assays showed that, as compared to control wild type (WT) cells, FAK-deficient cells were significantly more susceptible to parasite invasion. Lysosome spreading and a disarranged actin cytoskeleton, two features associated with susceptibility to MT invasion, were detected in FAK-deficient cells, as opposed to WT cells that exhibited a more organized F-actin arrangement, and lysosomes concentrated in the perinuclear area. As compared to WT cells, the capacity of FAK-deficient cells to bind a recombinant protein based on gp82, the MT surface molecule that mediates invasion, was higher. On the other hand, when treated with FAK-specific inhibitor PF573228, WT cells exhibited a dense meshwork of actin filaments, lysosome accumulation around the nucleus, and had increased resistance to MT invasion. In cells treated with PF573228, the phosphorylation levels of FAK were reduced and, as a consequence of FAK inactivation, diminished phosphorylation of extracellular signal-regulated protein kinases (ERK1/2) was observed. Fibronectin, known to impair MT invasion, induced the formation of thick bundles of F-actin and ERK1/2 dephosphorylation.

## Introduction

Focal adhesion kinase (FAK), a cytoplasmic protein tyrosine kinase (PTK) involved in the control of several biological processes, regulates the flow of signals from the extracellular matrix (ECM) to the actin cytoskeleton (Parsons et al., 2000). Cell adhesion to ECM proteins, such as fibronectin, triggers integrin-mediated signaling events, including the increased tyrosine phosphorylation of FAK, and stimulation of the mitogen-activated protein kinase ERK2 (Schlaepfer and Hunter, 1997). FAK has been shown to play a role in the internalization of pathogenic bacteria, such as *Salmonella typhimurium, Staphylococcus aureus, Escherichia coli*, and *Neisseria meningitidis* (Reddy et al., 2000; Agerer et al., 2005; Shi and Casanova, 2006; Slanina et al., 2012). The formation of focal adhesion-like complexes induced at sites of *S. typhimurium* attachment, and the dramatic impairment of bacterial uptake by FAK-depleted cells, demonstrated that FAK is required (Shi and Casanova, 2006). *S. aureus*, which triggered the recruitment of focal contact associated proteins, including FAK, to sites of bacterial attachment, had its internalization severely impaired in FAK-deficient cells (Agerer et al., 2005). Invasive *E. coli* K1 induced tyrosine phosphorylation of human brain microvascular endothelial cells FAK, which was recruited to focal plaques at the site of bacterial entry (Reddy et al., 2000). Treatment of target cells with specific FAK inhibitor reduced *N. meningitidis* internalization by more than 90% (Slanina et al., 2012).

The involvement of host cell PTK in the invasion process of *T. cruzi*, the protozoan parasite that causes Chagas disease, was first shown in macrophages by using PTK inhibitor genistein (Vieira et al., 1994). In non-phagocytic cells, such as human epithelial HeLa cells and rat kidney fibroblasts, genistein treatment had no effect on invasion by metacyclic trypomastigotes (MT), corresponding to the insect stage parasite forms, or by tissue culture-derived trypomastigotes (TCT), which are equivalent to parasite forms that circulate in the mammalian host bloodstream (Rodríguez et al., 1995; Neira et al., 2002). More recently, FAK was reported to play a role in TCT invasion of cardiomyocytes (Melo et al., 2014). Whether FAK is implicated in MT internalization remains to be investigated.

*Trypanosoma cruzi* MT invasion, which is mediated by the stage-specific surface glycoprotein gp82, relies on the host cell F-actin disruption, and lysosome spreading that culminates in exocytosis (Cortez et al., 2006; Martins et al., 2011). In this study, we generated FAK-depleted cells and determined the effect of FAK knockdown on F-actin organization, lysosome distribution, gp82 binding, and MT internalization. We also examined whether the treatment of wild type cells with FAK inhibitor PF573228 or fibronectin affected the actin cytoskeleton architecture, lysosome localization, and MT invasion. In addition, the phosphorylation profile of FAK and ERK1/2 was analyzed in wild type cells, either untreated or treated with FAK inhibitor or fibronectin, as well as in FAK-deficient cells.

## Materials and Methods

### Parasites, Mammalian Cells, and Cell Invasion Assay

*Trypanosoma cruzi* strain CL (DTU TcVI), derived from the vector *Triatoma infestans* in Rio Grande do Sul, Brazil (Brener and Chiari, 1963), was used throughout this study. Metacyclic forms of CL strain efficiently enter host cells mediated by gp82, which is the main MT surface molecule with cell adhesion property (Yoshida, 2006). For manipulation of parasites, a level 2 biosafety cabinet was used, in accord with the institutional safety recommendations (Certificate of Quality in Biosecurity (CQB) 028/97—Próton 6295/12). The parasites were grown in LIT medium and then cultured for one passage in Grace's medium (Thermo Fisher Scientific) to stimulate the differentiation of epimastigotes to metacyclic trypomastigotes, which were purified by passage through DEAE-cellulose column, as described (Teixeira and Yoshida, 1986). Maintenance of HeLa cells and MT invasion assays were performed as detailed, using MOI = 10 (Rodrigues et al., 2017). For extracellular amastigote (EA) cell invasion assays, *T. cruzi* G strain (DTY TcI), isolated from opossum in Amazon, Brazil (Yoshida, 1983), was used because G strain EAs efficiently enter HeLa cells whereas EAs of CL strain invade cells very poorly (Fernandes and Mortara, 2004). The procedure to generate EA from TCT derived from Vero cells followed a previously described protocol (Bonfim-Melo et al., 2015). Target cells were incubated for 1 h with EA (MOI = 5), fixed and Giemsa-stained. The number of internalized parasites was counted in a total of 250 cells in duplicate coverslips.

### Antibodies and Reagents

Anti-LAMP2 (H4B4) antibody was from Developmental Studies Hybridoma Bank developed under the auspices of the NICHD and maintained by The University of Iowa, Department of Biology, Iowa City, IA 52242. Alexa Fluor 488 phalloidin or TRITC-phalloidin and Alexa Fluor 488-conjugated anti-mouse IgG were from Thermo Fisher Scientific. Human fibronectin was from Sigma/Merck. Antibodies for FAK, phospho-FAK (Tyr^397^), phospho-44/42 MAPK (Erk1/2) (Thr^202^/Tyr^204^), β-tubulin, and GAPDH were from Cell Signaling Technology.

### Establishment of HeLa Cell Lines Deficient in FAK by Lentiviral Transduction

For FAK knockdown, we followed a protocol modified from that described previously (Bonfim-Melo et al., 2015), using plasmids containing target FAK sequences (Sigma Aldrich/Merck, Cat No. TRCN0000196310, sequence 1: CCGGGATGTTGG TTTAAAGCGATTTCTCGAGAAATCGCTTTAAACCAACATCTTTTTTG, and TRCN0000121318, sequence 2: CCGGCCGATTGGAAACCAACATATACTCGAGTATATGTTGGTTTCCAATCGGTTTTTG. Briefly, 3 ×10^6^ HEK293T cells were plated on 100 ×20 mm cell culture dishes (one dish per sequence) containing DMEM supplemented with 10% fetal bovine serum (FBS). After 24 h, HEK293T cells were transfected with calcium phosphate co-precipitation protocol, using 10 μg pCMV-dR8.91, 5 μg pVSVG, and 15 μg pLKO.1 (vector containing shRNA target sequence). The supernatant of cell culture, collected each 24 up to 72 h, was filtered in 0.45 μm syringe filter and was stored at −80°C until use or used immediately for HeLa transduction, which was performed in 6 well plates seeded with 4 ×10^4^ cells/well. Following addition to each well of 2 ml lentiviral preparation, in the presence of 4 μg/ml polybrene, and 24 h incubation, the medium was discarded and RPMI with 10% FBS (R10) was added. Twenty four hours later, the medium was replaced by R10 containing 0,2 μg/ml puromycin. The cells were maintained for 2 weeks, by changing medium each 2 or 3 days, with increasing concentrations of puromycin, up to 10 μg/ml, for selection of transduced cells. To check FAK depletion, the cells were disrupted with lysis buffer (50 mM tris-HCl, 150 mM NaCl, 1 mM EDTA, 1% Igepal CA630, protease inhibitor cocktail) and centrifuged at 13,400 × g for 10 min. Following the supernatant protein quantification, 40 μg protein was applied onto each lane of a 10% SDS-PAGE gel. Western blots were revealed with antibodies to FAK, to β-tubulin and/or GAPDH.

### Visualization of Actin Cytoskeleton and Lysosomes by Indirect Immunofluorescence

To visualize F-actin and lysosomes, HeLa cells grown on coverslips were fixed with 4% p-formaldehyde in PBS for 30 min at room temperature, HeLa cells were treated with 50 mM NH_4_Cl in PBS for 15 min, washed in PBS and then blocked for 1 h at room temperature in PGN-saponin (PBS containing 0.15% gelatin, 0.1% saponin, 0.1% sodium azide). Alexa Fluor 488 phalloidin or TRITC-phalloidin diluted 1:200 (v/v) in blocking solution plus 10 μg/ml DAPI (4′,6-diamidine-2′-phenylindole dihydrochloride), was used to detect F-actin and DNA, respectively. For lysosome visualization, the cells were processed as previously described, using anti-human LAMP2 antibody and Alexa Fluor 488-conjugated anti-mouse IgG (Rodrigues et al., 2017). Coverslips, mounted in ProLong Gold (Invitrogen) were analyzed at Leica TCS SP8 laser-scanning microscope (Leica, Germany), Instituto de Farmacologia e Biologia Molecular (INFAR), Universidade Federal de São Paulo, using Plan-Apochromat oil immersion and 63X objective. Images were processed and analyzed using Leica LAS AF (Leica, Germany) and Imaris (Bitplane) software.

### Recombinant Protein Gp82 (r-gp82) Production and Cell Binding Assay

The production and purification of r-gp82, containing the full-length *T. cruzi* gp82 sequence in frame with glutathione S-transferase (GenBank^TM^ data base, accession number L14824), followed the procedure described elsewhere (Cortez et al., 2006). Assay for cell binding of r-gp82 was performed essentially as described (Rodrigues et al., 2019).

### Statistical Analysis

The Student's *t* test, as implemented in GraphPad Prism software (Version 6.01), was used.

## Results

### Depletion of FAK Increases Host Cell Susceptibility to *T. cruzi* MT Invasion Mediated by Gp82

**Figure 1:**
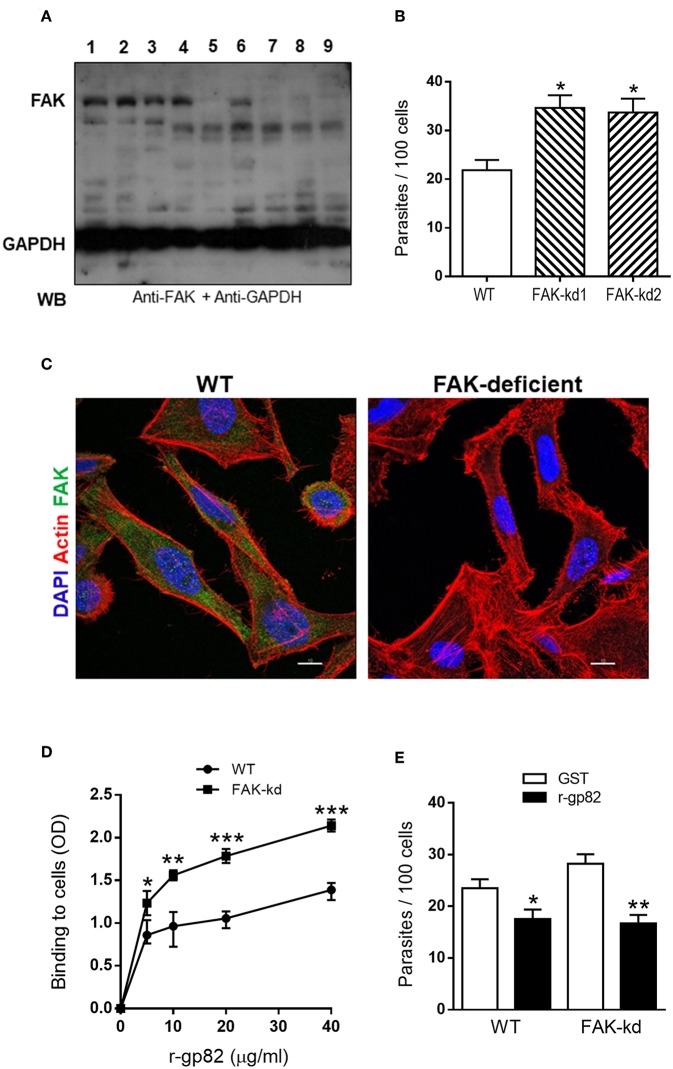
FAK knockdown increases host cell susceptibility to *T. cruzi* MT invasion and gp82 binding capacity. **(A)** HeLa cells were subjected to lentiviral transduction for FAK knockdown (kd) and then analyzed by western blotting (WB) using the indicated antibodies. Shown are the wild type (WT) cells (lane 1), cells transduced with lentivirus containing unrelated sequence (lanes 2 and 3), cells targeted with FAK sequence 1 (lanes 4, 5, 6) or with FAK sequence 2 (lanes, 7, 8, 9). Note the FAK depletion in four cell lines (lanes 5, 7, 8, 9). **(B)** WT cells and two FAK-depleted cell lines (FAK-kd1 and FAK-kd2) were incubated with MT for 1 h and then processed for intracellular parasite counting. Values are the means ± SD of three independent assays performed in duplicate. MT invasion was significantly increased in FAK-deficient cells (*P < 0.005). **(C)** WT and FAK-deficient cells were processed for immunofluorescence analysis using anti-FAK antibody, Alexa Fluor 488-conjugated anti-mouse IgG (green), TRITC-phalloidin (red) for actin detection and DAPI (blue) for DNA, under 63x objective. Scale bar = 10 μm. **(D)** WT and FAK-deficient cells, grown in microtiter plates, were fixed and incubated sequentially with r-gp82, anti-gp82 antiserum, and anti-mouse IgG conjugated to peroxidase. The binding of r-gp82, revealed with *o*-phenylenediamine and expressed as optical density value, was significantly higher in FAK-deficient cells than in WT cells (*P < 0.05, **P < 0.01, ***P < 0.001). A representative result of three independent assays performed in triplicate is shown. **(E)** WT and FAK-deficient cells were incubated with MT in the presence of r-gp82 or GST. Values are the means ± SD of three independent assays performed in duplicate. MT invasion was significantly decreased in the presence of r-gp82 (*P < 0.05, **P < 0.005).

To deplete FAK in HeLa cells by lentiviral transduction methodology, two different target sequences were used. Cells were also transduced with lentivirus containing an unrelated sequence. Wild type (WT) cells and puromycin-selected cells were analyzed by western blot using anti-FAK antibody. FAK was detected in WT cells (Figure 1A, lane 1) and in cells targeted with unrelated sequence (lanes 2 and 3). Of the cell lines targeted with FAK sequence 1 (lanes 4, 5, and 6) or FAK sequence 2 (lanes 7, 8, and 9), four were found to be depleted in FAK (lanes 5, 7, 8, and 9), two of which (lanes 5 and 7), designated as FAK-kd1 and FAK-kd2, were further amplified for experiments of MT invasion. WT and FAK-depleted cells were incubated with MT for 1 h and processed for intracellular parasite counting. Both FAK-depleted cells were significantly more susceptible to MT invasion, as compared to WT cells (Figure 1B). In all subsequent experiments, we used FAK-kd1 cells that were henceforth designated as FAK-deficient cells. Deficiency in FAK was also checked and confirmed by confocal microscopy analysis. WT and FAK-depleted cells were processed for immunofluorescence, using anti-FAK antibody. In WT cells, FAK was clearly visualized in the cytoplasm but it was undetectable (Figure 1C) or barely detectable (Figure S1) in FAK-depleted cells. We examined the ability of gp82, the MT surface molecule that mediates invasion, in binding to FAK-deficient cells. ELISA assay was performed in microtiter plates coated with WT or FAK-deficient cells, using the recombinant gp82 protein (r-gp82) at varying concentrations. Binding of r-gp82 was higher in FAK-deficient cells and displayed the same receptor-dependent profile of WT cells (Figure 1D). This result suggests that the increased *T. cruzi* MT internalization in FAK-deficient cells is due to an increased gp82 binding to its receptor, which was recently identified to be the lysosome membrane-associated protein LAMP-2 (Rodrigues et al., 2019). Experiments were performed to demonstrate that MT invasion of FAK-deficient cells is gp82-dependent. WT and FAK-depleted cells were incubated with MT in the presence of r-gp82 or GST, at 40 μg/ml. As shown in Figure 1E, MT internalization of FAK-depleted cells was significantly lower in the presence of r-gp82 than in the presence of GST.

### FAK-Deficient Cells Differ From WT Cells in Actin Cytoskeleton Architecture and Lysosome Distribution

**Figure 2:**
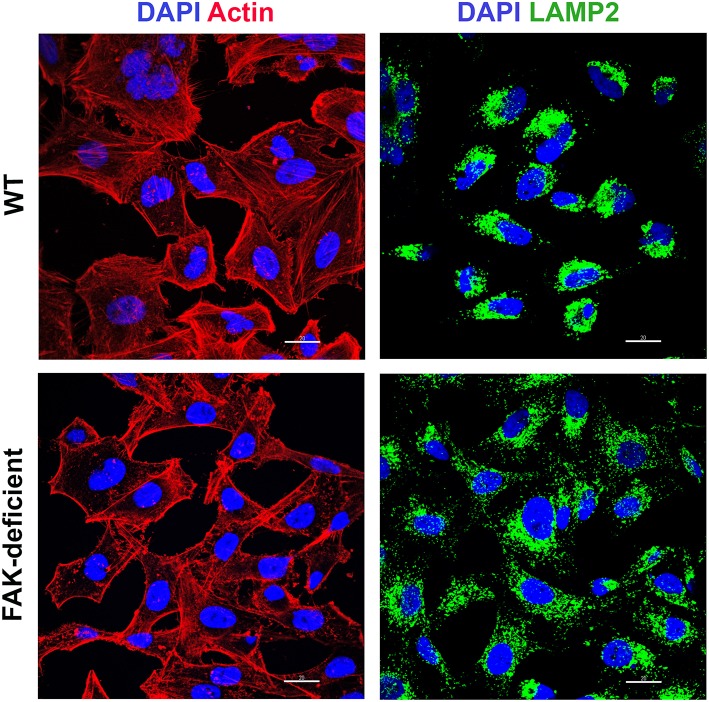
FAK-deficient and WT cells exhibit distinct F-actin organization and lysosome distribution. WT and FAK-deficient cells were analyzed by confocal fluorescence microscopy, to visualize actin cytoskeleton (red), lysosomes (green), and nucleus stained with DAPI (blue), under 63x objective. Scale bar = 20 μm. Note the F-actin disarrangement and lysosome spreading in FAK-deficient cells.

As HeLa cells are more susceptible to MT invasion under conditions in which the actin cytoskeleton is disrupted (Cortez et al., 2006) and lysosomes are spread to the cell periphery (Cortez et al., 2016), we compared the F-actin arrangement and the distribution of lysosomes in WT and FAK-depleted cells. Confocal microscopy analysis showed disassembled F-actin and lysosome spreading in FAK-deficient cells, as opposed to WT cells that displayed a more organized actin cytoskeleton and accumulation of lysosomes in the perinuclear region (Figure 2).

### Decrease in MT Entry Into WT Cells by FAK Inhibition Is Associated With ERK1/2 Dephosphorylation and F-actin Rearrangement

**Figure 3:**
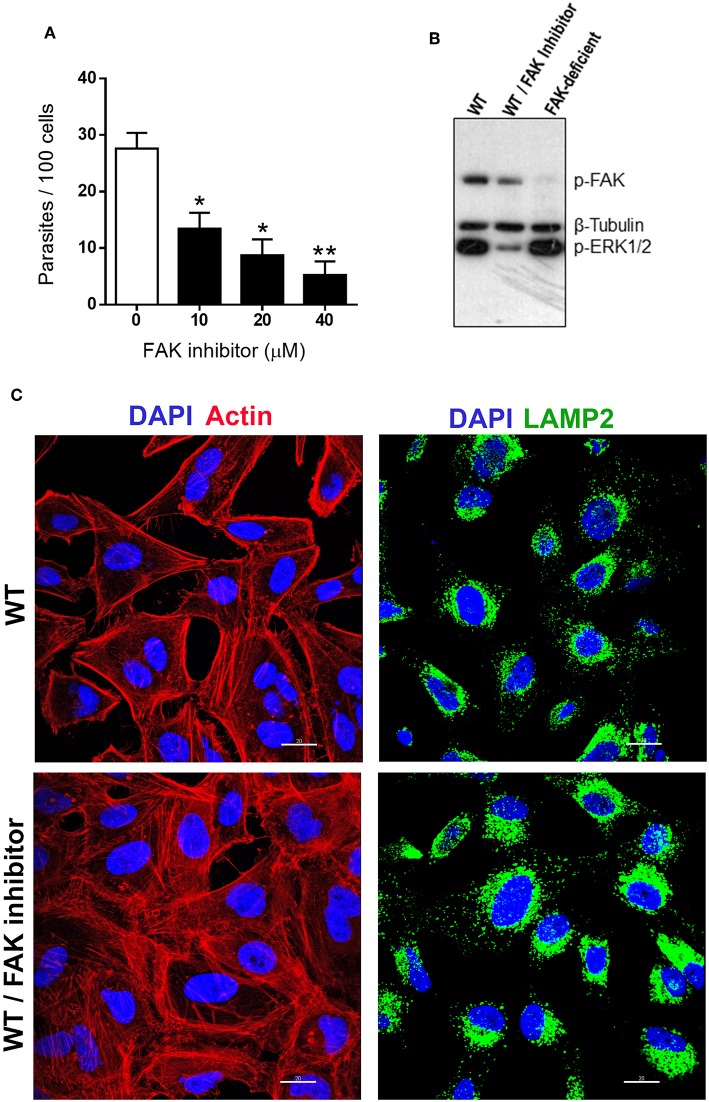
FAK inhibition in WT cells induces FAK and ERK1/2 dephosphorylation, and reduces MT invasion by affecting the actin cytoskeleton architecture. **(A)** Cells were untreated or treated for 45 min with FAK inhibitor PF573228 at the indicated concentrations, the drug was removed, parasites were added, and incubation proceeded for 1 h before intracellular MT counting. Values are the means ± SD of three independent assays performed in duplicate. MT invasion was significantly reduced in cells treated with FAK inhibitor (*P < 0.005, **P < 0.001). **(B)** Detergent-soluble extracts of untreated and FAK inhibitor-treated WT cells, as well as of FAK-deficient cells, were analyzed by western blotting, using antibody to phospho-FAK (Tyr^397^), phospho-ERK1/2 and β-tubulin. Note the decreased phosphorylation levels of FAK and ERK1/2 in FAK inhibitor-treated WT cells. **(C)** WT cells, treated or not with FAK inhibitor PF573228 and then processed for confocal fluorescence microscopy to visualize actin cytoskeleton (red), lysosomes (green), and nucleus (blue). Scale bar = 20 μm. Note the altered F-actin arrangement in FAK inhibitor-treated cells.

Although the experiments with FAK-deficient cells indicated that FAK is not essential for MT internalization (Figure 1B), we analyzed the effect of treatment of WT cells with specific FAK inhibitor PF573228. HeLa cells were either untreated or treated for 45 min with FAK inhibitor at various concentrations, in serum-free medium. After removal of the drug, the cells were incubated with MT for 1 h in complete medium and processed for intracellular parasite counting. The number of internalized MT was significantly lower in PF573228-treated cells at all concentrations, with >80% inhibition at 40 μM (Figure 3A). To ascertain that PF573228 inhibited FAK activity, leading to ERK1/2 dephosphorylation, we examined by western blot the phosphorylation status of FAK and ERK1/2 in HeLa cells untreated or treated with PF573228 at 40 μM, as well as in FAK-deficient cells, using antibody to phospho-FAK(Tyr^397^), phospho-ERK1/2 and β-tubulin, which served as loading control. Phosphorylation levels of FAK and ERK1/2 were reduced in WT cells treated with PF573228 (Figure 3B). In FAK-deficient cells, the phosphorylation of FAK(Tyr^397^) was barely detectable, as expected, whereas the profile of ERK1/2 phosphorylation was comparable to that of WT cells (Figure 3B). This result suggested that ERK/1/2 activation, which can be induced by a FAK-independent signaling, is associated with susceptibility to MT invasion. Experiments were performed to determine whether FAK inhibition affected the F-actin arrangement and lysosome localization in WT cells. After treatment with PF573228, the cells were processed for immunofluorescence, along with untreated controls. The architecture of actin cytoskeleton was altered in PF573228-treated cells, a dense meshwork of F-actin quite distinct from the untreated control being visualized, whereas the perinuclear lysosome accumulation was similar in both cells (Figure 3C).

### Fibronectin-Induced Decrease in MT Internalization Is Associated With ERK1/2 Dephosphorylation and F-actin Rearrangement

**Figure 4:**
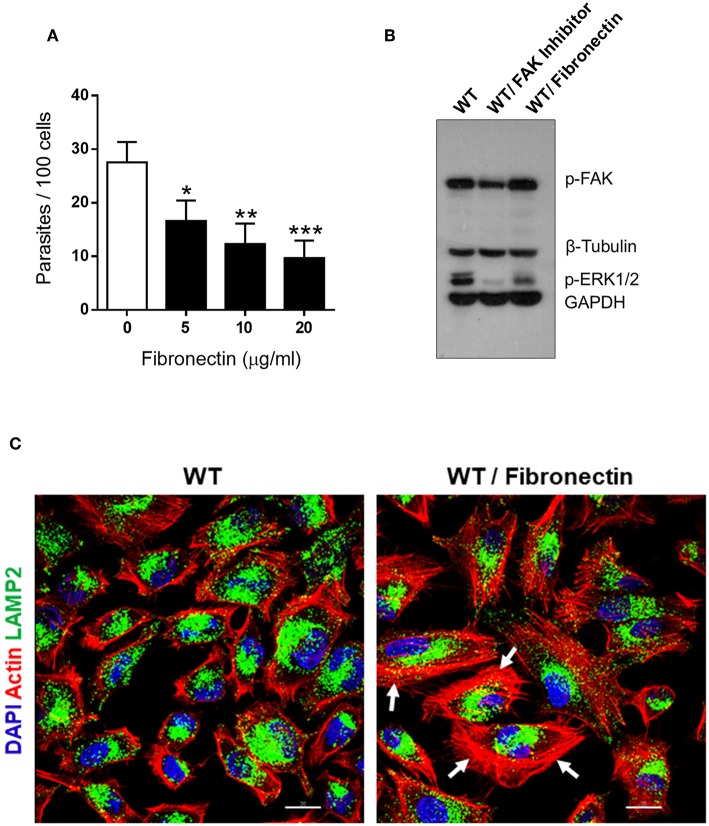
Fibronectin-induced decrease in MT invasion is associated with ERK1/2 dephosphorylation and F-actin bundle formation. **(A)** WT cells were pretreated for 30 min with fibronectin at the indicated concentrations, parasites were then added and the incubation proceeded for 1 h. After fixation and Giemsa staining, the internalized parasites were quantified in at least 250 cells/duplicate. Values are the means ± SD of four independent assays. MT invasion was significantly reduced in cells treated with fibronectin at all concentrations (*P < 0.01, **P < 0.005, ***P < 0.001). **(B)** Detergent-soluble extracts of cells untreated or treated with FAK inhibitor or fibronectin were analyzed by western blotting, using antibody to phospho-FAK (Tyr^397^), phospho-ERK1/2. Specific antibodies were used to reveal the loading controls β-tubulin and GAPDH. Note the decreased phosphorylation levels of ERK1/2, but not of FAK, in fibronectin-treated WT cells. **(C)** WT cells, treated or not with fibronectin, were processed for confocal microscopy to visualize actin cytoskeleton (red), lysosomes (green), and nucleus (blue). Scale bar = 20 μm. Note the dense F-actin bundles in fibronectin-treated cells (white arrows).

A previous study had shown that fibronectin impairs MT internalization (Maeda et al., 2014). Here we performed experiments to determine the effect of fibronectin on FAK and ERK1/2 activation, as well as on F-actin organization and lysosome localization. First, we checked the inhibitory effect of fibronectin on MT invasion. WT cells were incubated for 30 min with fibronectin at various concentrations, and then the parasites were added without removing fibronectin. After 1 h incubation, the number of intracellular parasites was counted. A significant inhibition of MT internalization was detected at all fibronectin concentrations (Figure 4A). Invasion assays, in which fibronectin was removed before incubation with MT, gave essentially the same results. Next, we examined by western blot the phosphorylation status of FAK and ERK1/2 in cells treated for 30 min in absence or in the presence of 20 μg/ml fibronectin. The phosphorylation levels of FAK(Tyr^397^) were similar in fibronectin-treated and untreated cells whereas those of ERK1/2 were diminished (Figure 4B). Confocal microscopy analysis revealed the formation of thick actin filament bundles in fibronectin-treated cells, not observed in control cells (Figure 4C).

### FAK Depletion or FAK Inhibition in WT Cells Does Not Affect Extracellular Amastigote (EA) Internalization

**Figure 5:**
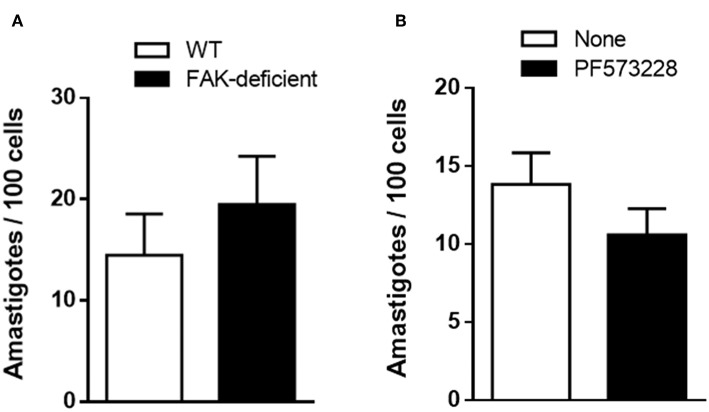
EA internalization is not significantly affected by FAK depletion or by treatment of WT cells with FAK inhibitor. **(A)** WT and FAK-deficient cells were incubated for 1 h with EA. **(B)** WT cells were treated or not with FAK inhibitor before incubation with EA. Intracellular parasites were quantified in at least 250 cells/duplicate. Values are the means ± SD of three independent assays. No significant difference was detected between FAK-deficient or FAK inhibitor-treated cells and the respective controls.

In addition to MT and TCT, amastigotes that may originate either from bloodstream trypomastigotes or after premature release from infected cells, can also invade cells (Hudson et al., 1984; Ley et al., 1988). We examined whether FAK depletion interfered with EA internalization, by incubating the parasites with WT and FAK-deficient cells for 1 h. An apparently increased susceptibility of FAK-deficient cells to EA invasion was observed, but it was not statistically significant (Figure 5A). Next, WT cells were treated or not with 40 μM FAK inhibitor PF573228 before incubation with EA. There was an apparent reduction of EA invasion in PF573228-treated cells, but it was not statistically significant (Figure 5B).

## Discussion

Our study has shown that cells depleted in FAK are more susceptible to *T. cruzi* MT invasion. A disorganized actin cytoskeleton and spreading of lysosomes, characteristics associated with higher susceptibility to MT invasion (Cortez et al., 2006; Martins et al., 2011), were observed in FAK-deficient cells. As the MT entry into cells is not impaired by FAK deficiency, it is clear that this tyrosine kinase is not required for the invasion process. However, the inhibition of FAK activity in WT cells, leading to decreased phosphorylation levels of FAK(Tyr^397^) as well as of ERK1/2, resulted in reduced MT internalization, suggesting that FAK is implicated. FAK-deficient cells exhibited highly phosphorylated ERK1/2, a profile similar to that of WT cells, suggesting the involvement of an alternative signaling pathway, independent of FAK.

FAK regulates the F-actin dynamics (Parsons et al., 2000; Li et al., 2013). Accordingly, we observed in FAK-deficient cells a more disorganized F-actin arrangement. Also observed was the spreading of lysosomes in the cytoplasm, compatible with earlier studies indicating that the disruption of actin cytoskeleton, which acts as a barrier for lysosome mobilization, facilitates its access to *T. cruzi* invasion site (Rodríguez et al., 1995, 1999). In WT cells, the inhibition of FAK led to the formation of highly bundled actin filaments, and higher resistance to MT invasion. Thick F-actin bundles were also detected in WT cells upon treatment with fibronectin, a procedure that reduces MT internalization. Cells treated either with FAK inhibitor or with fibronectin displayed lysosomes concentrated in the perinuclear region, like non-treated cells. The ERK1/2 phosphorylation levels were lower than in non-treated controls, what is compatible with the finding that the phosphorylation profile of ERK1/2 was similar in WT cells and FAK-deficient cells, both susceptible to MT invasion. The phosphorylation status of FAK(Tyr^397^) in fibronectin-treated cells was similar to that of non-treated controls, further supporting the notion that ERK1/2 activation may occur in FAK-independent manner. It was suggested in a previous study that binding of MT surface molecule gp82 to fibronectin hampered its recognition by the receptor engaged in the internalization process (Maeda et al., 2014). This and the fibronectin-induced F-actin rearrangement may both contribute for the resistance to MT entry into target cells. It is known that the link of ECM to the cytoplasmic actin cytoskeleton may be elicited by interaction of integrin receptors with extracellular ligands, including fibronectin (Parsons et al., 2000). In gp82-mediated MT internalization, there is no evidence that integrin receptor is implicated (Maeda et al., 2014). Rather than plasma membrane proteins, LAMP-2 was identified as gp82 receptor (Rodrigues et al., 2019).

Our data differ from those described for TCT invasion of cardiomyocytes, which was shown to be FAK-dependent. In contrast to the increased susceptibility of FAK-depleted cells to invasion by MT, the suppression of FAK in siRNA-treated cardiomyocytes increased the resistance to TCT internalization (Melo et al., 2014). This is consistent with the fact that conditions that promote lysosome spreading increased HeLa cell susceptibility to MT and resistance to TCT (Cortez et al., 2016). However, similarly to MT internalization, TCT invasion diminished upon treatment of cardiomyocytes with specific FAK inhibitor PF573228 (Melo et al., 2014), possibly because TCT internalization is favored by F-actin disruption (Rodríguez et al., 1995). As regards EA internalization, FAK depletion or FAK inhibition in WT cells had no significant effect. These parasite forms enter non-phagocytic target cells in a manner distinct from MT or TCT. A characteristic of EA-HeLa cell interaction is the formation of actin-enriched cup-like structures at parasite invasion sites (Procópio et al., 1998; Bonfim-Melo et al., 2018).

Taken together, our data have indicated that FAK is not required for MT invasion process. However, in WT cells the evidences are that the signaling pathway involving this tyrosine kinase is triggered during MT internalization. If FAK is inhibited, changes in the actin cytoskeleton structure, concomitant with perinuclear accumulation of lysosomes, render the cells more resistant to MT. Similar changes, induced by fibronectin and without alteration in FAK phosphorylation, had the same deleterious effect on MT invasion.

## Data Availability

All datasets generated for this study are included in the manuscript and/or the Supplementary Files.

## Author Contributions

NY and TO conceived and designed the experiments. TO and JR performed the experiments. TO, JR, and NY interpreted results. NY wrote the manuscript.

### Conflict of Interest Statement

The authors declare that the research was conducted in the absence of any commercial or financial relationships that could be construed as a potential conflict of interest.

## Supplementary Material

Figure S1 FAK is barely detectable in cells submitted to FAK depletion. WT and FAK-depleted cells were processed for immunofluorescence, as in Figure 1C. Scale bar = 10 μm. Note that in FAK-depleted cells reaction with anti-FAK antibody is minimal.
