# Supplementary figures and images for: Depletion of Host Cell Focal Adhesion Kinase Increases the Susceptibility to Invasion by Trypanosoma cruzi Metacyclic Forms
Source: Front Cell Infect Microbiol. 2019 Jun 26;9:231. doi: 10.3389/fcimb.2019.00231 (PMC6607697; doi:10.3389/fcimb.2019.00231)

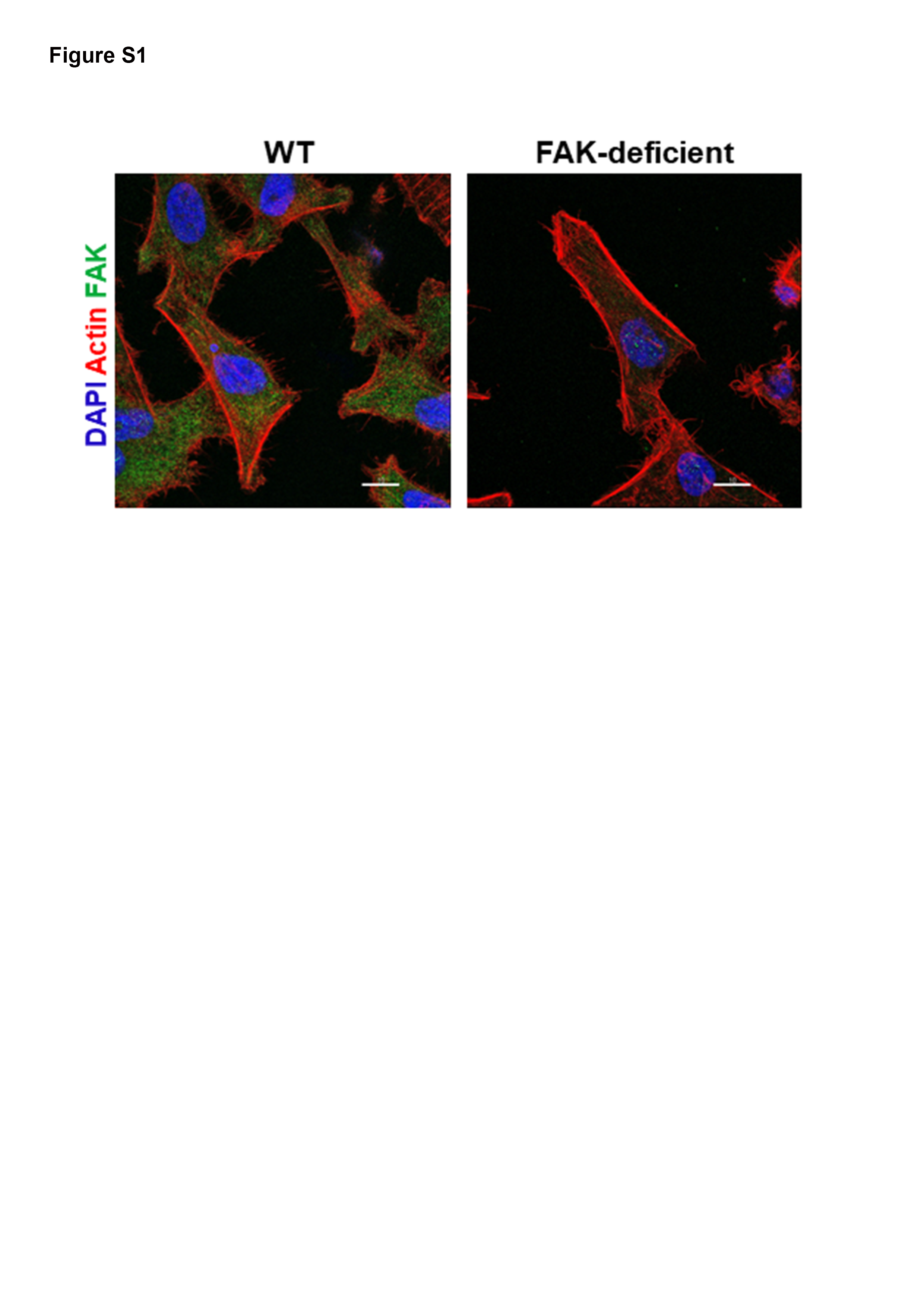

Supplement: Figure S1 — FAK is barely detectable in cells submitted to FAK depletion. WT and FAK-depleted cells were processed for immunofluorescence, as in Figure 1C. Scale bar = 10 μm. Note that in FAK-depleted cells reaction with anti-FAK antibody is minimal. [file Image_1.TIF]
